# Mental-Map Preserving Visualisation of Partitioned Networks in Vanted

**DOI:** 10.1515/jib-2019-0026

**Published:** 2019-06-14

**Authors:** Dimitar Garkov, Karsten Klein, Christian Klukas, Falk Schreiber

**Affiliations:** Department of Computer and Information Science, University of Konstanz, 78464 Konstanz, Germany; Digitalization of Research and Development, BASF SE, 67056 Ludwigshafen am Rhein, Germany; Faculty of Information Technology, Monash University, Clayton, Victoria 3800, Australia

**Keywords:** Network visualization, Network analysis, Mental map preservation, Data clustering, Graph drawing

## Abstract

Biological networks can be large and complex, often consisting of different sub-networks or parts. Separation of networks into parts, network partitioning and layouts of overview and sub-graphs are of importance for understandable visualisations of those networks. This article presents *NetPartVis* to visualise non-overlapping clusters or partitions of graphs in the Vanted framework based on a method for laying out overview graph and several sub-graphs (partitions) in a coordinated, mental-map preserving way.

## 1 Introduction

**Figure 1:**
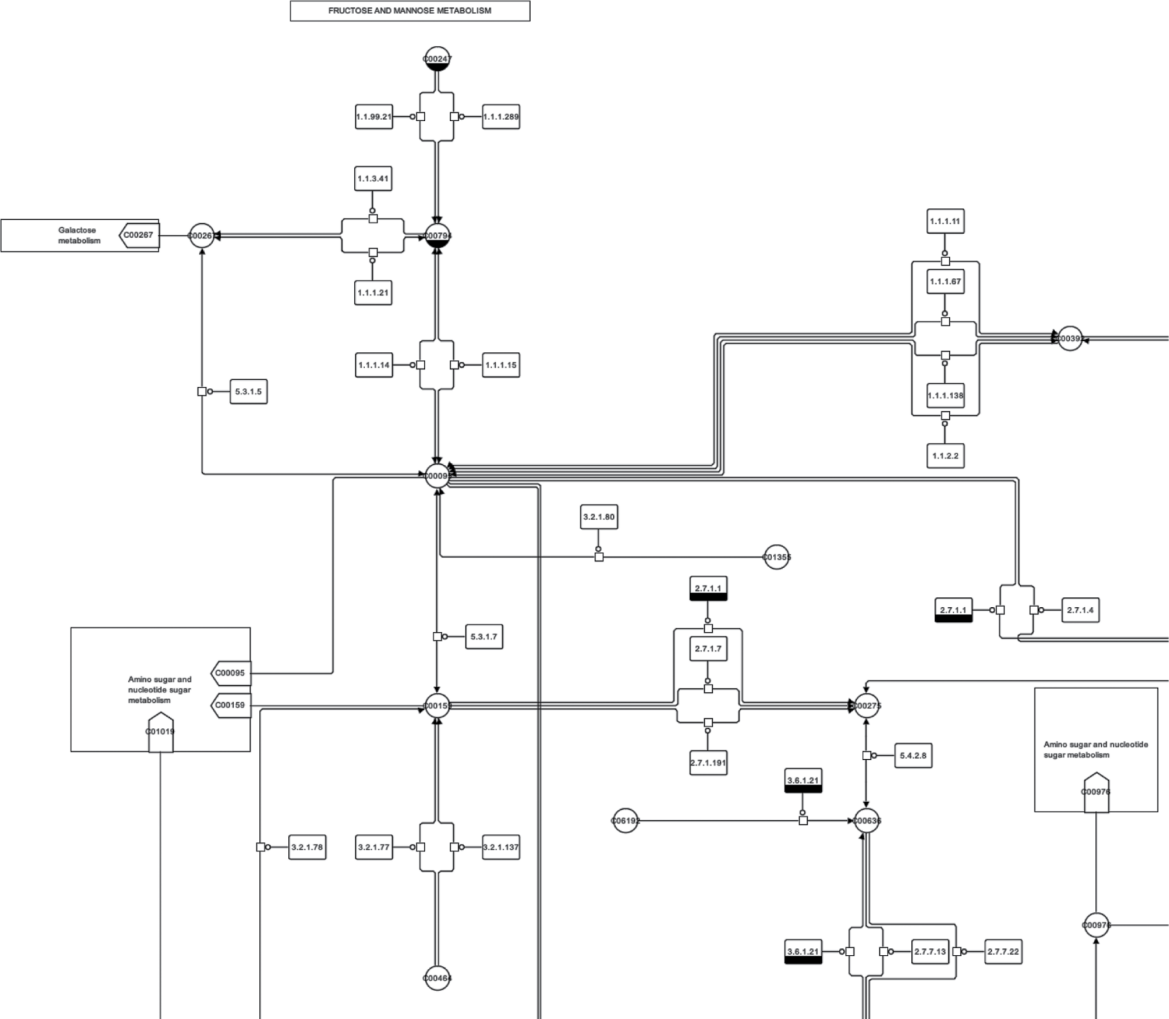
Typical visualisation of a metabolic network, in this case a cutoff of a metabolic pathway derived from KEGG [1]. The network is shown in the SBGN [2] representation computed by SBGN-ED [3], including automatic layout based on the original KEGG layout [4].

Biological systems are characterised by complex, interwoven processes which comprise thousands of elements such as different genes, transcripts, proteins and metabolites. There are different representations of these processes, for example, textual descriptions or mathematical equation systems. Often biological systems are also represented as networks or graphs, see Figure 1. More details regarding the representation of biological networks as graphs, typical layout methods for different biological networks as well as generic layout methods can be found in articles such as [5], [6], [7], [8].

**Figure 2:**
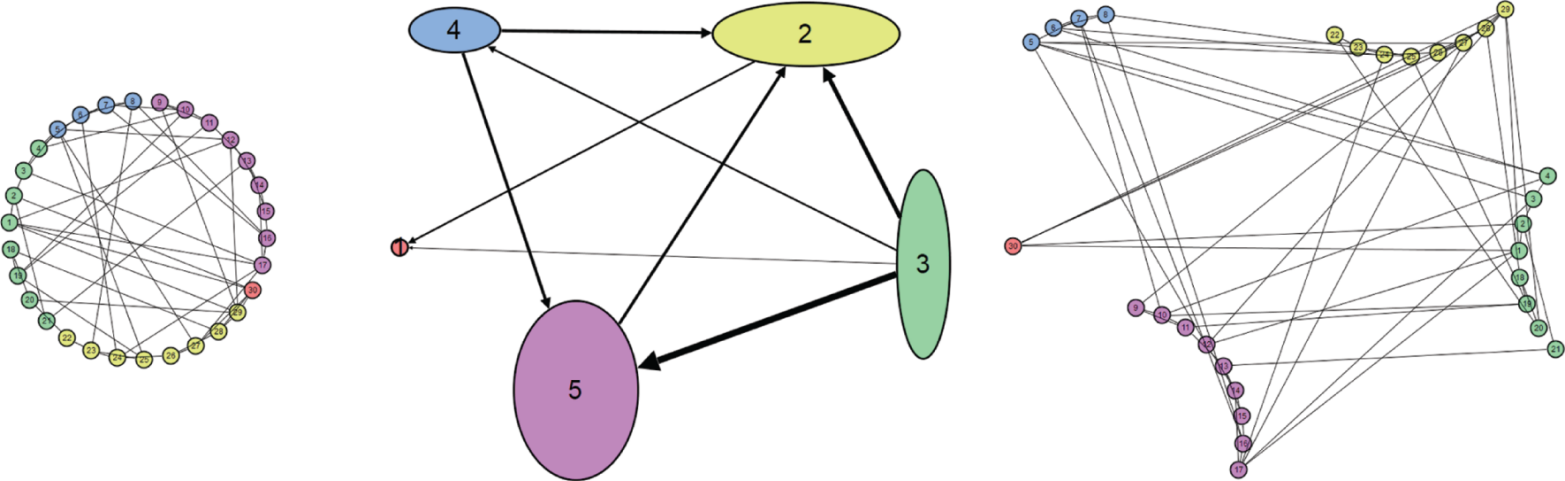
Graph *G* (with clusters marked by different colours), overview graph *G_O_* with one node for each cluster (in the respective colour) and mental-map preserving layout of *G* (preserving the structure of the overview graph within the graph *G*), in this case also without re-layout of the different clustered sub-graphs (from left to right).

Biological networks can be separated or partitioned in different ways. For example, there exists a large collection of network analysis algorithms including many graph clustering methods [9], [10], partly also for the computation of non-overlapping clusters (partitions). It is also possible to separate the highly interconnected processes into different process classes, such as gene regulatory processes, protein interaction and metabolic processes among several other processes. Independent of the base for partitioning, the result are several non-overlapping clusters in the graph, as well as an overview graph where each node represents a cluster of the graph. This can be formalised as follows:

A (non-overlapping) clustered graph *C* = (*G*, *G_O_*) consists of a base graph *G* and an overview graph *G_O_*, such that the nodes of *G_O_* represent the clusters of *G*, each node of *G* belongs to exactly one cluster, and there is an edge between nodes in *G_O_* if there are edges between nodes in the respective clusters in *G*, see Figure 2. Note that there are several definitions of clustered graphs, for example, a tree-based definition given by Eades defines a clustered graph *C* = (*G*, *T*) as consisting of a base graph *G* and a rooted tree *T*, such that the leaves of *T* are exactly the nodes of *G* [11]. Let *G* = (*V_G_*, *E_G_*) be the base graph. The hierarchy is defined by the tree *T* = (*V_T_*, *E_T_*), with the leaves *L*(*T*) = *V_G_*. A view is defined as a subset of *V_T_* that induces a partition of *V_G_* [12]. In the remainder of this article we will use the simple definition *C* = (*G*, *G_O_*) with an overview graph. Here we also do not want to distinguish between clustered and heterogeneous graphs [13] and therefore consider heterogeneous graphs also clustered graphs where, for example, each cluster contains a specific graph type (graph types can be undirected graphs, directed graphs or graphs with specific node types such as to represent metabolic networks, to name a few examples).

Graphs are a mathematical concept for expressing structural relationships between elements of a certain system. An adequate layout of a graph helps to visually recognise its substructures. Layout and visualisation of clustered graphs (including graph structures such a planar clustered graphs and compound graphs) have been a research area in graph drawing for more than 30 years. Early work includes multi-level visualisation of clustered graphs [11], clustering and visual abstraction [14], structured layouts that separate zones for sub-graphs [15], orthogonal grid drawings of clustered graphs [16], drawing of compound graphs [17] and convex drawings of planar clustered graphs [18]. While there have been approaches to apply the planarisation concept to clustered graphs in order to minimise crossings [19], [20], the complexity of the general decision problem is still unknown [21], and current solutions for sub-classes of clustered graphs are far from practical application [22]. There also exist specific approaches for clustered biological networks such as the layout of biological compound graphs [23]. To some extent visualisation solutions which compare several graphs and build a comparison tree [24] can also be seen as layouts of clustered graphs.

Mental-map preserving layouts, introduced by Misue et al. [25], play an important role for the understanding of layout changes. The mental map of a graph is the abstract representation of this graph in the user’s brain which is then used to quickly navigate through the graph visualisation when changes occur. Here we will use the term *mental-map preserving* for coordinated views where the layout of the overview graph is easily visible in the spatial relations of the different clusters – the mental map regarding the overview graph is preserved.

Vanted [26] is an open source framework for the analysis and visualisation of biological networks and related experimental data. An Add-on mechanism allows for simple extension of the Vanted core, and several extensions exist, for instance, to compute network centralities [27] and to visualise fluxes in networks [28]. Vanted supports the visualisation of all kinds of biological networks and supports graphical standards for biological networks, in particular SBGN PD [29], SBGN ER [30] and SBGN AF [31]. A typical workflow in Vanted is presented in [32] – in this workflow metabolic maps in the SBGN standard are constructed, enriched with different kinds of ^∗^omics data and exported to clickable websites. Other workflows can be found in [33].

The visualisation and exploration of networks is an important part of the Vanted workflow, therefore suitable methods to support these tasks are needed. Here we present *NetPartVis*, a method for layout and explorative visualisation of clustered graphs in Vanted which enables users to lay out an overview graph and the comprising sub-graphs (partitions) in a coordinated, mental-map preserving way. Due to the broad usability of such visualisations we decided to add *NetPartVis* to the main functionality of Vanted (core), therefore no additional loading of an Add-on is necessary.

## 2 *NetPartVis* – Layout and Visualisation of Clustered Graphs in Vanted

### 2.1 Construction of Graphs

Let *G* be the source (base) graph, *G_O_* the overview graph (initially empty) and *G*_1_, … , *G_k_* a set of graphs representing the clusters 1, …, *k*. Each node of *G* contains an ID (integer) from 1, …, *k*, representing the cluster this node belongs to.

The method for mental-map preserving visualisation of partitioned networks is based on the following data property: The source graph contains nodes that all have cluster IDs assigned. An overview graph and clustered sub-graphs are created from the source graph as follows:

1. For each distinct cluster ID that is assigned to at least one node in the source graph *G*, a new node representing a cluster is created in the overview graph *G_O_*.
2. For each edge in the source graph *G*, a new edge connecting nodes in *G_O_* is created, if and only if the cluster IDs of the source and target nodes of that edge in *G* are different. To avoid duplicated connections of nodes in the overview graph *G_O_*, in case of an existing edge no new edge is created, but a dedicated edge attribute, *edgecount*, is increased by one. Thereby the information on the number of connections between clusters in the source graph *G* remains available in the overview graph *G_O_*.
3. The sub-graphs *G*_1_, … , *G_k_* are then defined as follows: For *i* = 1, …, *k* graph *G_i_* contains all nodes with ID *i* and all edges, whose end nodes have cluster ID *i*.

To assign cluster IDs to nodes and edges in the source graph *G*, users are offered several options in Vanted. Users can

1. Enter a cluster ID for the current selection of graph elements;
2. Copy cluster IDs from the labels of the selected graph elements;
3. Let cluster IDs be determined from connected sub-graphs;
4. Sort graph elements into different clusters each with a distinct cluster ID, based on a given attribute, such as size, colour and position among others.

**Figure 3:**
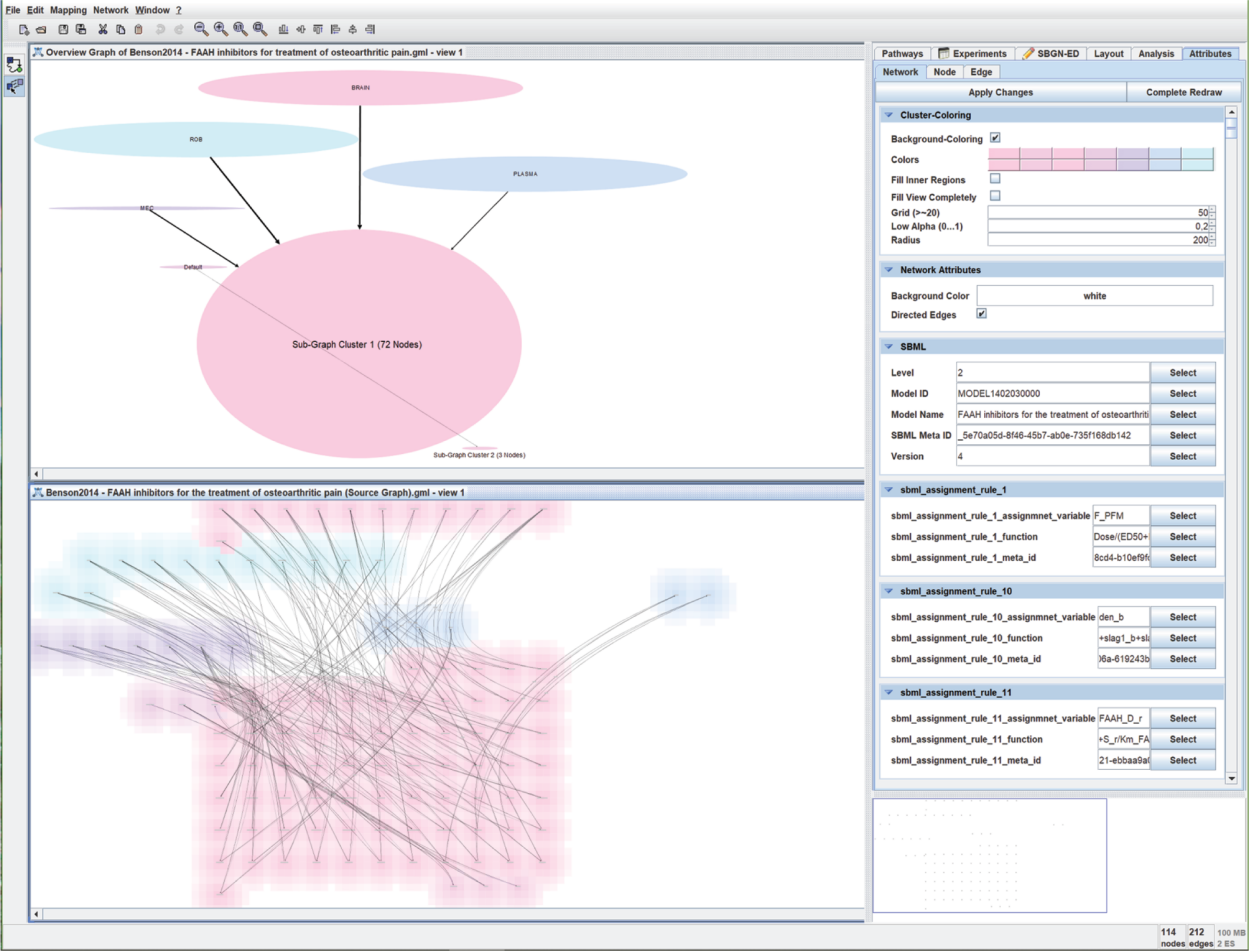
*NetPartVis* uses an overview graph *G_O_* (shown at the top, created from the source graph, shown at the bottom), to influence the placement of clusters in the source graph *G*. Here, the source graph has been downloaded from the BioModels database (https://www.ebi.ac.uk/biomodels/BIOMD0000000512) using Vanted 2.6.5. In addition to the pre-existing clusters given by BioModels (BRAIN, ROB, PLASMA, MEC and Default), the rest has been determined via the connected sub-graphs feature. Cluster background colouring (set from the Network Attributes Tab on the right) and edge bundling have been applied to the source graph.

To make different clusters better distinguishable in the source graph *G* and in the overview graph *G_O_*, nodes are colour-coded. Additionally, clusters can have their surrounding background also colour-coded. This can be particularly useful for very large clustered graphs. By changing the colour of the clusters, the colour of the corresponding nodes in the source and overview graphs, and the colour of the cluster backgrounds in the source graph, are modified, respectively. See Figure 3 for an example.

### 2.2 Layout of Graphs

To account for the size of the clusters and the connections between different clusters, the nodes and edges of the overview graph *G_O_* are modified:

1. For all nodes of *G_O_* holds: The size of a node *n_i_* in the overview graph is determined as described in the layout process below.
2. The thickness of an edge in *G_O_* is used to visualise the number of connections between clusters in the source graph *G* and for each edge of *G_O_* yields: The thickness of an edge is determined by its dedicated edge attribute *edgecount*.

**Figure 4:**
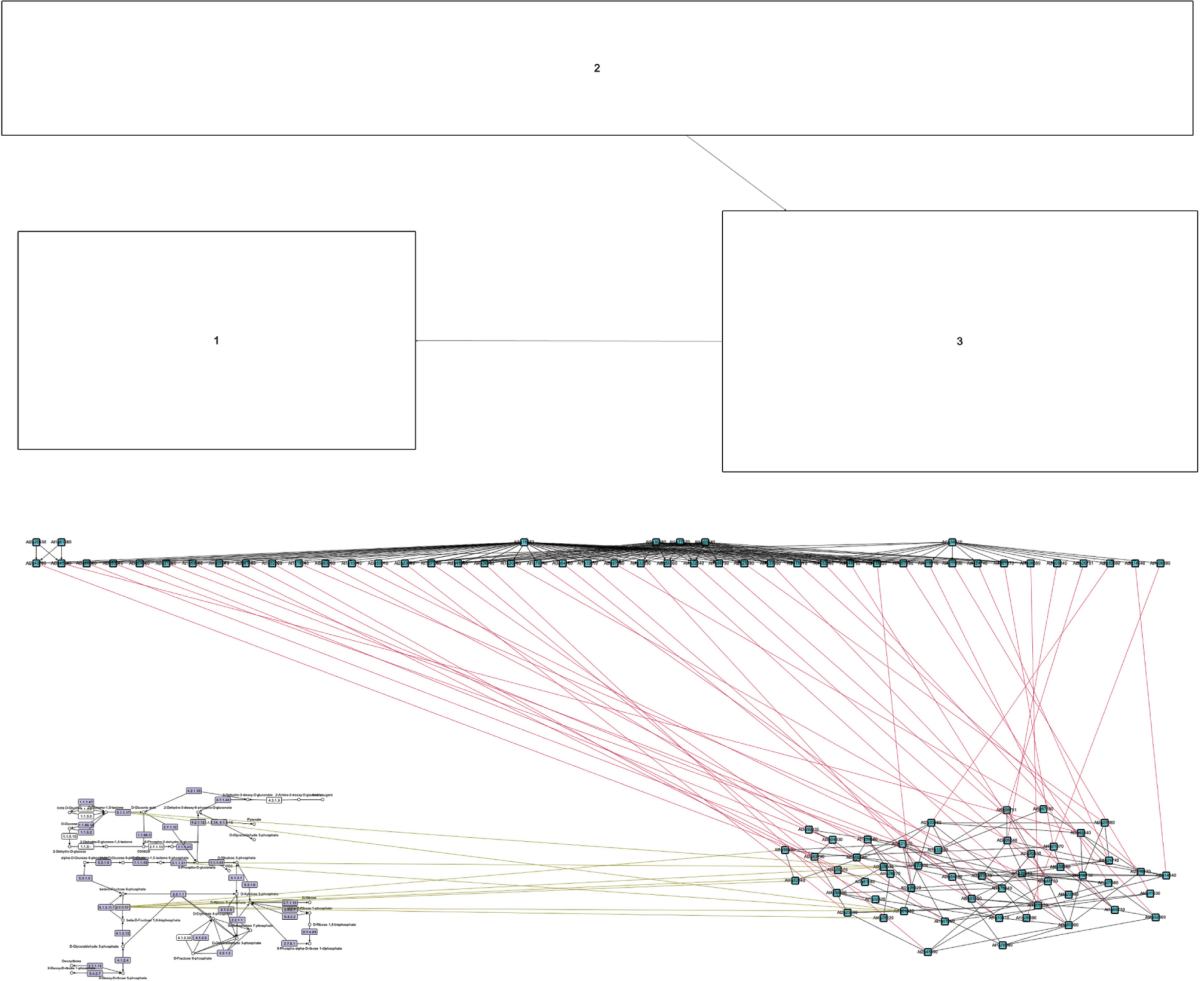
Overview graph (top) and clustered graph (bottom) for heterogeneous networks as an example. In contrast to previous figures where the size of nodes of the overview graph depends on the number of represented nodes, here the overview graph shows the bounding boxes of the layouts of the clustered sub-graphs.

The layout process consists of the following steps, illustrated in Figure 4:

1. Layout sub-graphs *G*_1_, … , *G_k_*: Any layout method available can be used, alternatively the existing layouts can be kept unmodified to preserve the layout of the clustered sub-graphs. The result of this step is that each sub-graph is laid out. Finally for each graph *G*_1_, … , *G_k_* the size of the bounding box is computed, and the size of the nodes *n*_1_, … , *n_k_* of *G_O_* is set to the size of the bounding box of the respective graph.
2. Layout overview graph *G_O_*: Any layout method available can be used. As the size of the nodes in *G_O_* represents the bounding box of the clustered sub-graphs, it is advisable that the layout takes node size into account or that the distance between nodes is large enough such that nodes do not overlap.
3. The positions of the nodes *n*_1_, … , *n_k_* of *G_O_* are used to layout *G*. The coordinates of nodes in *G* are based on the position of the nodes in *G_O_* and an offset based on the layout of the graphs *G*_1_, … , *G_k_*. For node *n* with cluster ID *i* the position is given by the position of *n_i_* of *G_O_* plus the position of *n* in *G_i_*.

Thanks to the framework implementation of Vanted, *NetPartVis* provides additional features to work with and analyse graphs, such as to select and modify nodes which belong to a given cluster or set of clusters. All discussed methods are available in Vanted (http://www.vanted.org).

## 3 Conclusion

We presented a method for visualising complex large graphs by graph partitioning and laying out an overview graph and several sub-graphs (partitions) in a coordinated, mental-map preserving way. *NetPartVis* is part of the Vanted system for the analysis and visualisation of experimental data in the context of biological networks. However, Vanted is also a general graph editor which can be used for graphs or networks from many other domains. Networks can be imported using several standards, such as GML [34], GraphML [35], SBGN-ML [36] and others, and be exported in the respective format, or alternatively as images and clickable web pages. Thus, the presented method, as well as its implementation is of broad use for network visualisation and exploration.
